# Predictors of Consanguinity Marriage Decision in Saudi Arabia: A Pilot Study

**DOI:** 10.3390/healthcare11131925

**Published:** 2023-07-03

**Authors:** Huny Bakry, Reema A. Alaiban, Alanood A. Alkhyyat, Basma H. Alshamrani, Rafal N. Naitah, Fatmah Almoayad

**Affiliations:** Department of Health Sciences, College of Health and Rehabilitation Sciences, Princess Nourah Bint Abdulrahman University, P.O. Box 84428, Riyadh 11671, Saudi Arabia; hmbakry@pnu.edu.sa (H.B.)

**Keywords:** consanguinity marriage, blood genetic disorders, marriage decision, Saudi Arabia

## Abstract

Consanguineous marriages are common in Saudi Arabia, increasing the risk of genetic blood disorders in offspring. This pilot study assessed the knowledge and perceived threats regarding genetic blood disorders, norms, and premarital screening for consanguineous marriage among unmarried university students in Saudi Arabia as a predictor of marriage decisions. A cross-sectional study was conducted from 22 January through 22 April 2022. In total, 400 unmarried students at Saudi Arabian universities were recruited using the non-probability convenience sampling technique. The data were analyzed using descriptive statistics and multinomial logistic regression. The results showed that the majority of participants had poor knowledge of genetic blood disorders. Most of the participants had a favorable attitude toward consanguineous marriage, while perceived threats towards genetic blood disorders were perceived as neutral by the participants. Moreover, their norms regarding consanguineous marriage also showed neutral results. A multinomial regression shows that participants with poor attitudes were significantly 22.3 times more likely to have poor marriage decisions (95% CI: 4.9–101.7, *p* < 0.001). However, participants with good and neutral norms regarding consanguinity marriage were significantly protective factors against poor marriage decisions with an RRR ratio of 0.165 (95% CI:0.030–0.918, *p* = 0.04) and 0.238 (95% CI: 0.071–0.797, *p* = 0.02), respectively. To mitigate the risk of genetic blood disorders in future generations, there is a need for targeted awareness campaigns about genetic blood disorders and the risks of consanguineous marriages by integrating this education into university curricula, and premarital counseling. It is also important to address societal norms, promoting informed decision-making, and provide premarital consultation to couples who carry the same mutated genes and are at risk of transmitting the disease to their offspring. Furthermore, there is a need for further research to assess the effectiveness of campaigns in this regard.

## 1. Introduction

Genetic blood disorders encompass a range of inherited conditions resulting from chromosomal abnormalities that negatively affect red blood cell (RBC) function [1]. Thalassemia and Sickle Cell Disease (SCD) are among the most prevalent genetic blood disorders, impairing the normal functioning of RBCs [2]. The risk of transmitting these diseases among parents who carry the same mutated gene is a 25% chance of developing the disease and a 50% chance of becoming carriers of the mutated gene [3].

One of the most important risk factors contributing to the spread of genetic blood diseases is consanguineous marriage, which constitutes 20–50% of marriages in North Africa, the Middle East, and West Asia [4]. Primary prevention of genetic blood disorders includes pre-marital screenings that identify genes responsible for these conditions, thus detecting high-risk couples [2]. Many underlying reasons encourage consanguineous marriages, most of which are economic or social beliefs [4]. Social norms are an important factor that plays a central role in the practice of consanguineous marriage; they highly influence families and can cause changes in marriage decisions [4]. Internationally, about 7% of the total population are carriers for genetic blood disorders, and 30,000–500,000 children are born with Sickle Cell disease (SCD) and thalassemia [5].

The Kingdom of Saudi Arabia (KSA) is a country with a high rate of genetic disorders. To the best of our knowledge, the latest statistics were updated in 2004–2009. According to the pre-marital examination, the national statistics of people who suffered from thalassemia were 1033, with a rate of 0.05%, and 45,892 were carriers of the disease, with a rate of 1.4% [1,5]. As for SCD, about 4.2% of the Saudi population were carriers, and about 0.27% suffered from the disease [6]. Therefore, in Saudi Arabia, premarital screening has been offered to reduce the spread of genetic blood diseases. This premarital screening program has been successful in reaching the target population, identifying high-risk couples, and reducing the number of high-risk marriages. However, there is still a significant number of couples who proceed with marriage despite being identified as high-risk, indicating the need for further measures [7]. Previous research has been conducted in Saudi Arabia to quantify levels of knowledge about SCD [8]. The results indicated that 71.2% of the participants had poor knowledge, and 59% had poor attitudes toward SCD.

The statistics show a critical challenge in Saudi society—the high prevalence of genetic disorders, mainly due to consanguinity. Despite efforts through premarital screening programs to combat these disorders, there are a surprising number of couples who still choose to marry, knowing the genetic risks involved. This intriguing reality raises significant questions about the attitudes, beliefs, and knowledge driving these decisions. To investigate deeper into this issue, it is crucial to adopt a comprehensive behavioral lens, focusing on the determinants of health behavior. Hence, to guide this research, two theoretical frameworks were adopted: the Theory of Planned Behavior (TPB) and the Health Belief Model (HBM). These theories provide robust tools to understand and analyze the decision-making process surrounding consanguineous marriages, particularly in light of potential genetic risks.

TPB is one of the most influential and widely used psychological theories to explain and predict behaviors. This theory states that change is achieved and maintained when a person intends to adopt a positive behavior or stop a negative one. According to TPB, intention is how a person will behave in a goal-directed way; a person’s intent is what makes them start to implement a certain behavior. Many studies have demonstrated that knowledge and perception also influence intention and behavior [9]. According to TPB, an intention to act is affected by norms and attitudes. Studying them is very important to know which factors are stronger in affecting an intention to practice [9], and when predicted, they can help guide health promotion programs. The theory suggests that intention is driven by attitudes and subjective norms [9].

Attitude refers to how positively or negatively a person evaluates a specific behavior. Attitude towards a certain behavior is based on a belief that associates it with a particular outcome; it is formed automatically and simultaneously. Therefore, people are able to form favorable attitudes towards behaviors they believe will have desirable effects [9]. A study in Saudi Arabia indicated that 51.9% of participants did not favor consanguineous marriage. The study identified an association between educational sessions and attitude changes, highlighting the importance of providing health education [10]. Subjective norms determine whether or not the social environment approves of a behavior. A positive subjective norm is held by an individual who believes that those in the social environment support the execution of a certain action, motivating the individual to satisfy their expectations [11].

Moreover, HBM is used to understand why people may not adopt preventive measures. It posits that an individual’s perceived personal threat influences the likelihood of adopting a behavior. Perceived threat consists of severity and susceptibility combined [9]. Many studies on health behavior suggest that an increase in perceived threats leads to increased precautionary behaviors. However, people who have a high risk may still doubt their ability to take precautions or the efficacy of those precautions [12].

Finally, Knowledge can be both a prerequisite for action and a result of action [13]. A study conducted in the KSA showed that 71.2% of the participants had a poor level of knowledge regarding SCD. This might indicate the need for awareness campaigns [8].

Blood genetic disorders are highly prevalent in Saudi Arabia due to the elevated rate of consanguineous marriages. These disorders are continually increasing, placing KSA as one of the leading countries in the incidence of blood genetic diseases [14]. The issue with blood genetic disorders is that they are rapidly passed down through generations. Moreover, blood genetic disorders impact not only the physical and psychological health of patients but also their socio-economic aspects. Socially, the presence of a person with a blood genetic disorder in the family can heighten emotional stress and lead to financial instability [4]. Consequently, Saudi Arabia has initiated the Saudi Genome Programme, one of the national projects designed to achieve Saudi Vision 2030, with the aim of reducing blood genetic diseases through genome sequencing of the Saudi population [15]. Evaluating the knowledge and perceived threats regarding blood genetic disorders and assessing norms and attitudes towards consanguineous marriage among university-aged students who are future parents will help in identifying gaps, thereby providing a more comprehensive and clear understanding of this issue. In addition, it can inform public health programs that design interventions that influence marriage decisions.

This study aims to investigate the perceptions, attitudes, and understanding of unmarried university students in Saudi Arabia regarding consanguineous marriages and associated genetic blood disorders.

Objectives:

1. Assess the knowledge and perceived threats of unmarried university students in Saudi Arabia regarding genetic blood disorders.
2. Identify the norms and attitudes of unmarried university students in Saudi Arabia regarding consanguinity marriage.
3. Assess intended marriage decisions in relation to knowledge, perceived threats regarding genetic blood disorders, and norms and attitudes regarding consanguineous marriage.

## 2. Materials and Methods

### 2.1. Study Design

A cross-sectional pilot study was conducted among university students in Saudi Arabia over a period of three months, from 22 January 2022 to 22 April 2022. A cross sectional design was chosen for time and cost efficiency. A non-probability convenience sampling technique was used for recruiting participants, and data was collected via an online self-administered questionnaire. Participants were recruited through the university’s official email list and online platforms, such as social media. A non-probability convenience sampling technique was used for recruiting participants due to its accessibility and ease of implementation. However, it is acknowledged that this sampling technique may limit the generalizability of the findings.

### 2.2. Population and Inclusion Criteria

The study population included single Saudi university students. Selected as they represent a group at an important transitional stage in life when decisions about marriage and family planning are often made. Understanding the knowledge, attitudes, and norms of this population could help inform interventions targeting this group. The minimum sample size required was calculated to be 385, based on a total population greater than 10,000. This was with a degree of error of 5%, prevalence estimated at 50% as suggested by Macfarlane [16], confidence level of 1.96, and design effect of 1. The participants were recruited using a convenience sampling technique.

### 2.3. Research Instruments and Tools of the Study

The research tool used in this study was a questionnaire that consisted of 60 questions. A questionnaire was chosen as the research tool because it is a cost-effective and efficient method to collect data from a large number of participants and because it allows for standardized data collection. The design of the knowledge and perceived threat questions was guided by two previous tools. Nine questions were adopted from Miri-Moghaddam and colleagues [17], and three questions were adopted from Al-ghubishi and colleagues [18]. The researchers paraphrased the questions to suit the targeted population and created more questions to cover the research objectives. The questionnaire consisted of five sections.

Section 1. There were 10 sociodemographic questions regarding nationality, age, sex, marital status, academic status, academic level, type of university, specialty, type of residence, and residential area.

Section 2. There were 32 questions that covered knowledge regarding genetic blood disorders. The correct answers were coded as 1 and the wrong answers as 0, for a total possible score of 32. A cut-off point was taken at the median, which was 9. For the purpose of this study, a score of 9 or more was considered good knowledge, and a score below 9 was considered poor knowledge.

Section 3. There were six questions that covered perceived threats from genetic blood disorders. The answer choices were presented on a 3-point Likert scale; therefore, there was a total possible score of 18. The cut-off points were determined at quartiles one and three. A total score equal to or below 10 was considered low, 11–14 was interpreted as moderate, and a score of 15 or above was a high perceived threat of genetic blood disorders.

Section 4. There were two questions that assessed attitude using a 3-point Likert scale. The total possible score was 6. The cut-off points were determined at quartiles one and three. Attitude was considered poor if it was 4 or below, 5 for a moderate attitude, and 6 for a good attitude. Poor participants indicated participants who supported or preferred consanguineous marriage, whereas good participants indicated participants who opposed the consanguineous marriage and did not support it.

Section 5. There were two questions that assessed norms using a 3-point Likert scale. The total possible score was 6. The cut-off points were determined at quartiles one and three. A total norm score was considered poor if it was 4 or below, 5 for moderate, and 6 for good norms. Poor participants indicated participants who supported or preferred consanguineous marriage, whereas good participants represented participants who opposed the consanguineous marriage and did not support it. In addition, poor indicated a participant’s perception towards their family’s attitude in supporting consanguineous marriage, whereas good indicated a participant’s perception towards their family’s attitude in opposing consanguineous marriage and not supporting it.

Lastly, a question was included about the participant’s intended marriage decision when a couple’s premarital examination did not match. The question was presented as a multiple-choice question. The answers were categorized as follows: 3 for a good decision, 2 for neutrality, and 1 for a poor decision. ‘Poor’ represents participants who will continue the marriage regardless of the pre-marital screening result, whereas ‘good’ signifies participants who will seek genetic counseling to help them make the correct decision. ‘Neutral’ is for those who have no clear idea regarding their future decision.

### 2.4. Validity and Reliability

The questionnaire was validated by four experts in the field of public health. The tool was piloted among 40 individuals to test the feasibility and clarity of the questions, and no changes were needed. Reliability was assessed using Cronbach’s alpha test. The total knowledge showed reliability with a score of 0.8. Perceived threats regarding blood genetic disorders were 0.5 and 0.4 for the norms regarding consanguinity marriage; the values were acceptable except for the norms, which were not satisfactory, which is attributed to the low number of questions [19].

### 2.5. Data Analysis

The data were analyzed using JMP version 16.2 [20]. The data are presented in frequency tables as numbers and percentages. The associations between different variables were tested using the chi-square test. A multinomial logistic regression was used to predict factors that affect the intended decision towards consanguinity marriage when a couple’s results do not match. The chi-square test and multinomial logistic regression were selected due to their suitability for investigating associations between categorical variables and predicting factors influencing the intended decision toward consanguineous marriage. The chosen methods account for possible confounding factors and biases in the study design by controlling for pertinent sociodemographic variables.

### 2.6. Ethics Approval

The study was approved by the Institutional Review Board (IRB) at Princess Nourah Bint Abdulrahman University (PNU) (#22-0006). The participants were informed about the study’s aims and the expected time needed to complete the questionnaire. Participants were also assured of the confidentiality of their data. Participation was voluntary, and the anonymity of the participants was maintained.

## 3. Results

Table 1 presents the sociodemographic characteristics of the study sample. Female participants represented 75.25% of the study sample. Approximately 44.22% of the participants were 19 years old or younger. Regarding academic level and specialty, 71% were juniors, and 53.25% of the participants were from the College of Sciences. In addition, 77.25% of the participants were from urban areas, and 96.75% of them were attending governmental universities. Regarding the existence of genetic diseases, 83.8% of the participants did not know if they had any of the listed genetic diseases, 3.8% reported having thalassemia, 6.2% had SCD, and 6.2% had other genetic blood disorders. Regarding a family history of genetic disorders, 56.7% of the participants reported that their families did not have genetic blood disorders, 15.5% had one of the two diseases in their family, and 27.8% did not know if they had either of the diseases in their family or not. Finally, in the participants’ family history of consanguineous marriage, 74.2% reported that there was a consanguineous marriage in their family, 17.8% reported that there was not, and 8% did not know if there was a consanguineous marriage in their family or not.

Table 2 presents the participants’ knowledge of genetic blood disorders. Approximately 63.5% of the participants knew that SCD was a genetic disease, and 74% knew how SCD was diagnosed. However, only 39.7% knew how thalassemia was diagnosed. In addition, 89.7% of the participants incorrectly answered the question about migraines as a symptom of SCD, and 93.75% of the participants incorrectly answered the question about paralysis of the extremities as a complication of SCD. In contrast, 90.5% of the participants correctly answered the question about internal bleeding as a complication of SCD. With regard to thalassemia, only 9.5% correctly answered the question about apoplexy (organ haemorrhage) as a complication, and 84.7% answered incorrectly when asked if individuals with thalassemia need lifelong medication. In general, 54.7% of the participants had poor knowledge of genetic blood disorders, while 45.3% of the participants had good knowledge.

Table 3 presents the participants’ responses regarding the perceived threats of genetic blood disorders. Approximately 78.75% thought that complications of SCD were dangerous, and 48.75% agreed that complications of thalassemia were dangerous. In addition, 69.5% of the participants believed that consanguineous marriage played a role in SCD, compared to 41.3% for thalassemia. In regard to dealing with complications, 36% of the participants believed that the complications of SCD could be dealt with, compared to 22% for thalassemia. Finally, 50.7% of the participants had neutral total scores regarding perceived threats of genetic blood disorders, while 47% had high and 2.3% had low responses regarding perceived threats.

Table 4 presents the attitudes and norms of the studied sample towards consanguineous marriage. Approximately 43.5% of the participants had a neutral attitude, while 55% had a good attitude (consanguineous marriage), and 1.5% had a poor attitude (opposed consanguineous marriage). Regarding the norms, 48.5% of the participants had neutral responses to them, while 39% had good and 12.5% had poor responses to them.

Table 5 presents the statistics from the analysis of the association between the different variables and the sociodemographic factors. There was a significant association between knowledge about genetic blood disorders and gender (χ^2^ = 10.315, *p* < 0.001), and there was a significant association between knowledge and specialty (χ^2^ = 13.091, *p* = 0.004). There was a significant association between perceived threats and gender (χ^2^ =6.041, *p* = 0.04). There was also a significant association between gender and norms (χ^2^ =10.22, *p* = 0.006). Approximately 75.3% of the participants were female, and 46.2% had moderate norms toward genetic blood disorders. There was a significant association between attitude and age (χ^2^ = 11.090, *p* = 0.0256). There was another significant association between attitude and gender (χ^2^ =27.944, *p* < 0.0001).

The intention to complete marriage or not when the result of a pre-marital examination does not match was examined by one question; 76.4% of the participants answered that they would not complete the marriage when there was no agreement between the two parties as a result of the examination, while 4.8% answered that they would complete the marriage, and 18.8% were not sure what their decision would be.

Table 6 shows that participants with poor attitudes were significantly 22.3 times more likely to make poor marriage decisions (95% CI: 4.9–101.7, *p* < 0.001). Conversely, participants with good and neutral norms regarding consanguinity marriage were found to be significantly 0.165 and 0.238 times less likely to make poor marriage decisions (95% CI:0.030–0.918, *p* = 0.04) and (95% CI: 0.071–0.797, *p* = 0.02), respectively. Additionally, participants with high and neutral perceived threats regarding blood genetic disorders were significantly 0.027 and 0.032 times less likely to make poor marriage decisions (95% CI: 0.002–0.299, *p* = 0.003) and (95% CI: 0.003–0.358, *p* = 0.005), respectively. On the other hand, poor knowledge regarding blood genetic disorders was identified as a significant predictor for a neutral marriage decision, where participants with poor knowledge were significantly 2.164 times more likely to make a neutral marriage decision (95% CI: 1.12–4.181, *p* = 0.02). Furthermore, poor and neutral attitudes toward consanguinity marriage were significantly 4.652 and 2.9 times more likely to result in a neutral marriage decision (95% CI: 2.212–9.78, *p* < 0.001) and (95% CI: 1.448–5.808, *p* = 0.003), respectively. In addition, high and neutral perceived threats are 0.098 and 0.151 times less likely to result in a neutral marriage decision (95% CI: 0.018–0.535, *p* = 0.007) and (95% CI: 0.028–0.81, *p* = 0.027), respectively.

## 4. Discussion

This research assessed unmarried university students’ knowledge and perceived threats regarding genetic blood disorders, along with their attitudes and norms towards consanguineous marriage in Saudi Arabia. Finally, it examined their intended marriage decisions in relation to these different variables.

In this research, almost half of the participants showed poor knowledge of genetic blood disorders. Similarly, a study conducted in Riyadh about knowledge of SCD demonstrated that more than two-thirds of the participants had poor knowledge [8]. Another study conducted in Jeddah about knowledge of thalassemia revealed that more than half of the participants had poor knowledge [21]. This could be due to the lack of educational programs and campaigns regarding genetic blood disorders. As for gender, more than half of the female participants had good knowledge regarding genetic blood disorders, while more than one-quarter of the male participants had good knowledge. These findings are consistent with other studies conducted in Saudi Arabia, as a previous study in Jeddah showed that females had significantly higher knowledge of thalassemia [21]. While the current study and the Jeddah study sample are skewed towards females (75.3%, and 67%, respectively), a study conducted in Abha with a majority of male participants (61.5%) reported similar findings, where almost twice as many females as males had good knowledge regarding SCD [22]. Regarding marital status in the current study, those who were engaged had better knowledge than those who were not engaged or divorced; this might be because the engaged individuals were more interested in diseases that may affect their future offspring. Most of the participants who had good knowledge were from medical and science colleges. It is likely that they received more medical information during their courses, as revealed by the significant association between medical specialties and good knowledge of health. Future initiatives are needed to raise awareness and improve knowledge about genetic blood disorders, the risk associated with consanguineous marriages, their potential, and the potential effects on their children.

In this research, most of the participants perceived no threats regarding genetic blood disorders. There was a significant association between perceived threats and gender, where half of the females showed high perceived threats regarding genetic blood disorders, while a minority of male participants showed high perceived threats. A previous study showed that females are more risk-averse than males; this might be the reason why females have higher perceived threats than males [23]. It is thus important to raise awareness regarding the realistic risk of congenital diseases, especially in consanguineous marriages.

In this research, more than half of the participants opposed consanguineous marriage. This may be explained by the age of the participants in this study, as the current generation generally has a wider variety of choices and more experiences than past generations. Therefore, they may be more open to marriage to a non-relative. There was a significant association between attitudes toward consanguineous marriage and age. Almost half of the participants, who were 19 years old or younger, opposed consanguineous marriage. This could be due to the fact that a young generation usually reflects a greater awareness of adhering to societal norms. As for gender, it was significantly associated with attitudes towards consanguineous marriage. More females (more than half of the participants) had an opposing attitude than males. This could be due to the fact that the females had better knowledge than the males. Therefore, a better awareness of genetic blood disorders can have affected their views on consanguineous marriage. A study in Riyadh demonstrated that attitudes could be affected by the level of knowledge [24]. The same study showed that almost half of the participants did not favor consanguineous marriage, and those who did favor it were from an older age group, those married to a relative, and people who had a family history of consanguineous marriage. Their experience with consanguineous marriage seemed to have influenced their attitudes [24]. Obtaining medical information regarding consanguinity has been identified as a significant changeable risk factor affecting a negative attitude toward consanguinity [10]. It has also been proven that there is a significant association between attitude and the decision to opt for a consanguineous marriage [21].

In this research, a minority of the participants had poor norms towards consanguineous marriage, as they believed it was a family preference and would be encouraged even when pre-marital examination results did not match. This reflects an increasing awareness in recent years about consanguineous marriage and the ability to make a marriage decision regardless of the community’s norms.

There are several influences that were found to significantly affect marriage decisions. Poor attitude was a significant predictor of poor marriage decisions, potentially due to the fact that individuals with poor attitudes are more prone to thoughtlessness and making decisions based on feelings rather than logic. The current study also revealed that having good and neutral norms regarding consanguinity marriage can serve as a protective factor against making poor marriage decisions. This could be attributed to individuals with these norms being more aware of the risks of consanguinity marriage, which influences them to prioritize the well-being of their offspring when making choices. Furthermore, high and neutral perceived threats regarding blood genetic disorders were found to be protective against making poor marriage decisions. Individuals who perceive these threats are more likely to possess knowledge about the risks their child may face if they have a blood genetic disorder, and they may have concerns about their child’s ability to lead a normal life. On the other hand, a neutral marriage decision may indicate an unclear understanding of the risks associated with blood genetic disorders and their potential impact on their children.

A study was conducted to review the efficacy of the theory of planned behavior in explaining and predicting health-related behaviors. The conclusion was that this theory explains intentions very well [25]. Another study conducted in Riyadh demonstrated that family pressure, living near family, and traditions were the main causes of completing this type of marriage, even if the pre-marital test did not match [24].

The study is methodically sound, with a clear objective, a carefully calculated sample size, a comprehensive questionnaire validated by experts, and robust data analysis techniques. The limitations of this study were the convenience sampling technique, which affected the generalizability of the data. In addition, the data is skewed towards more female and younger respondents. While it is common to have more female respondents in online surveys [26], it does indeed limit the understanding of underrepresented groups.

## 5. Conclusions

In conclusion, this study examined the perceptions, attitudes, and understanding of unmarried university students in Saudi Arabia regarding consanguineous marriages and associated genetic blood disorders through three objectives. In regard to the first objective, the findings demonstrate that more than half of the participants (54.7%) have poor knowledge of genetic blood disorders. In addition, half of the participants (50.7%) had a neutral perception of the threats of SCD and thalassemia. Regarding the second objective, a good attitude towards the risk of consanguinity was found among more than half of the participants (55.0%), and the perception of norms was moderate among nearly half of the studied sample (48.5%). The last objective, which assessed the marriage decisions, revealed that 76.4% of the participants would not proceed with the marriage if the pre-marital examination results were incompatible. The study found that a poor attitude toward consanguinity marriage was a significant predictor of poor marriage decisions. However, good norms toward consanguinity marriage and high perceived threats regarding blood genetic disorders were protective against making poor marriage decisions.

These findings emphasize the need for targeted awareness campaigns. These campaigns should focus on improving knowledge about genetic blood disorders, the risk associated with consanguineous marriages, and their potential impact on offspring. These programs could be implemented as part of the elective university curriculum to reach the intended target population. Given that those engaged were more knowledgeable, targeted interventions for soon-to-be married couples could be part of premarital counseling services. This could help raise awareness about genetic disorders and the risk of consanguineous marriage and promote informed decision-making. The research also highlighted that the perceived threats of genetic blood disorders were neutral among participants, suggesting the need to clarify the realistic risk of these diseases, especially in the context of consanguineous marriages. Gender differences in knowledge and perceived threats also suggest the need for gender-specific educational initiatives targeting men. While the majority of participants opposed consanguineous marriage, a substantial number held moderate or favorable views. Interventions that address societal norms around consanguineous marriage and raise awareness about the associated risks may be beneficial. Moreover, there was a relationship between these variables and the intention towards a marriage decision, suggesting these initiatives could play a crucial role in shaping future family planning decisions. Furthermore, given the relationship between knowledge, attitudes, and intended decisions around marriage, premarital consultation should be provided to high-risk couples following the premarital screening. This would ensure that they have all the information they need to make informed decisions about their future, bearing in mind the potential transmission of genetic blood disorders across generations. Further research needs to be conducted with engaged couples and an assessment of the effectiveness of genetic disorder awareness campaigns.

## Figures and Tables

**Table 1:** General characteristics of the participants.

Variables	*n*	%
Age (*n* = 398)		
≤19	176	44.2%
20–21	147	36.2%
>21	75	18.6%
Gender (*n* = 400)		
Female	301	75.3%
Male	99	24.7%
Academic level (*n* = 400)		
Years 1–2	284	71%
Years 3–4	52	13%
Years 5–6	64	16%
Speciality (*n* = 398)		
College of Humanities	86	21.6%
College of Sciences	213	53.4%
College of Health Science	81	20.4%
Applied Colleges	18	4.6%
Type of university (*n* = 400)		
Governmental	387	96.7%
Private	13	3.3%
Residence (*n* = 400)		
Urban	309	77.3%
Rural	91	22.7%
Having a genetic blood disorder (*n* = 400)		
Thalassemia	15	3.8%
Sickle cell anemia	25	6.2%
Other genetic disorders	25	6.2%
Do not know	335	83.8%
Family history of genetic blood disorders (*n* = 400)		
No family history	227	56.7%
Have a genetic disorder in the family	62	15.5%
Do not know	111	27.8%
Family history of consanguinity marriage (*n* = 400)		
Yes	297	74.2%
No	71	17.8%
Do not know	32	8%

**Table 2:** Knowledge of participants about genetic blood disorders (*n* = 400).

Questions	Correct		Incorrect	
	*n*	%	*n*	%
Knowledge of studied participants about Sickle cell anemia				
1. Sickle cell anemia is a genetic disease	254	63.5%	146	36.5%
2. Symptoms of sickle cell anemia:				
Obesity	178	44.5%	222	55.5%
Migraine	41	10.3%	359	89.7%
Abdominal bloating and pain	132	33.0%	268	67.0%
Constant pain episodes	147	36.7%	253	63.3%
Increase sugar level in blood	86	21.5%	314	78.5%
Jaundice/yellowing of the skin	244	61.0%	156	39.0%
3. Sickle cell anemia patients are at risk of pain episodes when:				
low blood sugar	57	14.3%	343	85.7%
Exposure to extreme heat or cold	228	57.0%	172	43.0%
Low blood pressure	29	7.3%	371	92.7%
Intense exercising	186	46.5%	214	53.5%
4. Sickle cell anemia patients are more likely to get infectious diseases	183	45.7%	217	54.3%
5. Diagnosis of Sickle cell anemia	296	74.0%	104	26.0%
6. complications of Sickle cell anemia:				
loss of sight	86	21.5%	314	78.5%
Infertility and the inability to have children	88	22.0%	312	78.0%
Apoplexy	130	32.5%	270	67.5%
Internal bleeding	38	9.5%	362	90.5%
7. Sickle cell anemia patients require blood transfusions throughout their lives	88	22.0%	312	78.0%
8. Sickle cell anemia can be treated	130	32.5%	270	67.5%
9. Sickle cell anemia patients need lifelong medication	184	46.0%	216	54.0%
Knowledge of studied participants about Thalassemia				
1. Thalassemia is a genetic disease	100	25.0%	300	75.0%
2. Diagnosis of Thalassemia	159	39.7%	241	60.3%
3. Symptoms of Thalassemia:				
Osteoporosis	132	33.0%	268	67.0%
Weakness and shortness of breath	111	27.7%	289	72.3%
Redness of skin	37	9.3%	363	90.7%
Loss of sense of taste	54	13.5%	346	86.5%
4. Thalassemia can be cured by stem cell transplantation	101	25.3%	299	74.7%
5. Complications of Thalassemia:				
Apoplexy	38	9.5%	362	90.5%
Paralysis of the extremities	25	6.3%	375	93.7%
Slow growth	111	27.7%	289	72.3%
Change in the shape of the facial bones	87	21.7%	313	78.3%
6. Thalassemia patients need lifelong medication	61	15.3%	339	84.7%
Total knowledge	Good		Poor	
	181	45.3%	219	54.7%

**Table 3:** Perceived threats of genetic blood disorders among the studied participants (*n* = 400).

Questions	Agree		Neutral		Disagree	
	*n*	%	*n*	%	*n*	%
I think that complications of thalassemia are dangerous.	195	48.7%	180	45.0%	25	6.3%
I think that complications of sickle cell anemia are dangerous.	315	78.7%	76	19.0%	9	2.3%
I think that consanguinity marriage plays a role in sickle cell anemia.	278	69.5%	100	25.0%	22	5.5%
I think that consanguinity marriage plays a role in the transmission of thalassemia genes across generations.	165	41.3%	200	50.0%	35	8.7%
Complications of sickle cell anemia can be dealt with.	144	36.0%	184	46.0%	72	18.0%
Complications of thalassemia can be dealt with.	88	22.0%	235	58.7%	77	19.3%
Total perceived threats	High		Neutral		Low	
	188	47.0%	203	50.7%	9	2.3%

**Table 4:** Attitudes and norms of studied participants towards consanguinity marriage (*n* = 400).

Questions	Agree		Neutral		Disagree	
	*n*	%	*n*	%	*n*	%
Attitudes						
I prefer consanguineous marriage.	33	8.3%	123	30.7%	244	61.0%
It is important for a person with a blood disease to go to a genetic counselor before making a decision to have a baby.	330	82.5%	56	14.0%	14	3.5%
Norms						
My family prefers a consanguinity marriage.	117	29.3%	130	32.5%	153	38.2%
My family will support the marriage if the pre-marital examination result does not match.	41	10.3%	82	20.5%	277	69.2%
Total norms	Good		Neutral		Poor	
	156	39.0%	194	48.5%	50	12.5%
Total attitude	220	55.0%	174	43.5%	6	1.5%

**Table 5:** Association between different variables and sociodemographic factors (*n* = 400).

Variables	Knowledge		*p*	Perceived threats			*p*	Norms			*p*	Attitudes			*p*
	P	G		L	M	H		P	M	G		P	M	G	
	*n* %	*n* %		*n* %	*n* %	*n* %		*n* %	*n* %	*n* %		*n* %	*n* %	*n* %	
Age															
≤19	94 53.4%	82 46.6%	0.72	4 2.3%	93 52.8%	79 44.9%	0.67	20 11.3%	83 47.2%	73 41.5%	0.24	1 0.57%	69 39.20%	106 60.23	0.02 *
20–21	79 53.8%	68 46.2%		2 1.4%	71 48.3%	74 50.3%		14 9.5%	73 49.7%	60 40.8%		2 1.36%	62 42.18%	83 56.46%	
>21	44 58.7%	31 41.3%		3 4%	38 50.7%	34 45.3%		14 18.7%	38 50.7%	23 30.6%		3 4%	42 56%	30 40%	
Gender															
Female	151 50.1%	150 49.9%	0.001 *	6 2%	143 47.5%	152 50.5%	0.04 *	32 10.6%	139 46.2%	130 43.2%	0.006 *	3 1%	110 36.54%	188 62.46%	0.0001 *
Male	68 68.7%	31 31.3%		3 3%	60 60.6%	36 36.4%		18 18.2%	55 55.6%	26 26.2%		3 3.03%	64 64.65%	32 32.32%	
Academic level															
Junior	152 53.6%	132 46.4%	0.57	7 2.5%	146 51.4%	131 46.1%	0.93	33 11.6%	141 49.7%	110 38.7%	0.31	4 1.41%	113 39.79%	167 58.80%	0.07
Moderate	32 61.6%	20 38.4%		2 2%	45 46.1%	48 51.9%		5 9.6%	22 42.3%	25 48.1%		0 0%	26 50%	26 50%	
Senior	35 54.7%	29 45.3%		0 1.6%	12 51.5%	9 46.9%		12 18.8%	31 48.4%	21 32.8%		2 3.1%	35 54.7%	27 42.2%	
Specialty															
College of Humanities	58 67.4%	28 32.6%	0.004 *	3 3.5%	43 50%	40 46.5%	0.06	14 16.3%	41 47.7%	31 36%	0.20	1 1.16%	36 41.86%	49 56.98%	0.4
College of Sciences	115 53%	98 46%		3 1.4%	118 55.4%	92 43.2%		20 12.4%	109 46.9%	84 40.7%		3 1.41%	98 46.01%	112 52.58%	
College of Medicine Science	33 40.8%	48 59.2%		2 2.5%	29 35.8%	50 61.7%		10 12.4%	38 46.9%	33 40.7%		1 1.23%	28 34.57%	52 64.20%	
Applied Colleges	12 66.7%	6 33.3%		1 5.6%	11 61.1%	6 33.3%		5 27.8%	5 27.8%	8 44.4%		1 5.5%	10 55.6%	7 38.9%	
Type of university															
Governmental	212 54.8%	175 45.2%	0.95	9 2.3% 196	50.7%	182 47%	0.84	48 12.4%	187 48.3%	152 39.3%	0.81	5 1.3%	166 42.89%	216 55.81%	0.053
Private	7 53.9%	6 46.1%		0 0% 7	53.8%	6 46.2%		2 15.4%	7 53.9%	4 30.7%		1 7.69%	8 61.54%	4 30.77%	
Residence															
Urban	48 52.8%	43 47.2%	0.66	9 2.9%	162 52.4%	138 44.7%	0.08	35 11.3%	150 48.5%	124 40.1%	0.37	5 1.62%	136 44.01%	168 54.37%%	0.8
Rural	171 55.3%	138 44.7%		0 0%	41 45%	50 55%		15 16.5%	44 48.4%	32 35.1%		1 1.10%	38 41.76%	52 57.14%	

Note: P = poor; M = moderate; G = good; L = low; H = High. Significance * *p* < 0.05.

**Table 6:** Multinomial logistic regression of marriage decisions (*n* = 400).

		Exp(B)/RRR	95% Confidence Interval		Sig.
			Lower Bound	Upper Bound	
**Poor decision**	Attitude				
	Poor	22.334	4.904	101.718	0.987
	Neutral	2.664	0.391	18.145	0.000 ***
	Good				0.317
	Knowledge				
	poor	0.324	0.088	1.192	
	good				0.090
	Age				
	19	1.974	0.445	8.748	
	20	1.602	0.356	7.212	0.371
	22				0.539
	Gender				
	Male	1.229	0.333	4.531	
	Female				0.757
	Norms				
	Good	0.165	0.030	0.918	
	Neutral	0.238	0.071	0.797	0.040 *
	Poor				0.020 *
	Threats				
	High	0.027	0.002	0.299	
	Neutral	0.032	0.003	0.358	0.003 **
	Low				0.005 **
**Neutral decision**	Attitude				
	Poor	4.652	2.212	9.780	0.000 ***
	Neutral	2.900	1.448	5.808	0.003 **
	Good				
	Knowledge				
	poor	2.164	1.120	4.181	0.022 *
	good				
	Age				
	19	0.885	0.408	1.919	0.757
	20	0.937	0.443	1.980	0.864
	22				
	Gender				
	Male	0.536	0.282	1.018	0.057
	Female				
	Norms				
	Good	1.100	0.399	3.031	0.853
	Neutral	1.569	0.625	3.937	0.337
	Poor				
	Threats				
	High	0.098	0.018	0.535	0.007 **
	Neutral	0.151	0.028	0.810	0.027 *
	Low				

Note: RRR: Relative Risk Ratio Significance ***, *p* < 0.0001, **, *p* < 0.01, *, *p* < 0.05.

## Data Availability

The data presented in this study are openly available in FigShare at doi.10.6084/m9.figshare.23615649, [27].
